# Muscle pain induced by hypertonic saline in the knee extensors decreases single-limb isometric time to task failure

**DOI:** 10.1007/s00421-020-04425-2

**Published:** 2020-06-29

**Authors:** Samuel A. Smith, Dominic Micklewright, Samantha L. Winter, Alexis R. Mauger

**Affiliations:** 1 Endurance Research Group, School of Sport and Exercise Sciences, University of Kent, Chatham, Kent, ME4 4AG UK; 2 School of Sport, Rehabilitation and Exercise Sciences, University of Essex, Wivenhoe Park, Colchester, UK

**Keywords:** Endurance, Exercise-induced pain, Fatigue, Hypertonic saline, Isometric, Nociception

## Abstract

**Purpose:**

Increased nociceptive activity and the experience of exercise-induced pain (EIP) may contribute to fatigue during endurance exercise. To investigate this, a pain model that produces pain similar to EIP and decouples its relationship to exercise intensity is required. This study (1) compared the quality of pain caused by a hypertonic saline injection into the vastus lateralis in resting and exercise conditions, and (2) investigated whether this pain contributes to changes in time to task failure.

**Methods:**

On separate days, 18 participants completed a time to task failure at 20% maximal voluntary torque (MVT), a resting hypertonic saline intramuscular injection, and in a further three visits a time to task failure at 10% MVT following injection of isotonic saline, hypertonic saline or a control (no injection).

**Results:**

In a subset of eligible participants (*n* = 12), the hypertonic saline combined with 10% MVT produced a qualitative experience of pain (assessed by the McGill Pain Questionnaire) that felt similar to EIP. 10% MVT with hypertonic saline significantly elevated pain intensity in the first 20% of the time to task failure and caused a significantly (*P* < 0.05) shorter time to task failure (448 ± 240 s) compared with the isotonic saline (605 ± 285 s) and control (514 ± 197 s) conditions.

**Conclusion:**

These findings demonstrate that hypertonic saline increases the intensity of pain during exercise, which results in a faster occurrence of exercise-induced fatigue. These results provide important evidence supporting pain as a limiting factor in endurance performance.

## Introduction

Intense and prolonged muscle contractions result in acute pain proportional to the intensity and duration of exercise (Cook et al. 1997). This ‘exercise-induced pain’ (EIP) arises from the sensitisation and activation of ascending group III and IV nociceptive afferents in response to the accumulation of endogenous algesics and increases in noxious and mechanical pressure within the contracting skeletal musculature (O’Connor and Cook 1999). The experience of EIP is often accompanied by fatigue (Pollak et al. 2014), which is defined as an exercise-induced reduction in the capacity to produce muscle force or power (Bigland-Ritchie and Wood 1984). This association has led to the suggestion that EIP may accelerate fatigue development during intense and prolonged exercise (Mauger 2014).

In support of this notion, the stimulation of muscle nociceptors and increased muscle afferent activity has demonstrated significant reductions in voluntary activation of the elbow flexors (Kennedy et al. 2013) and maximal voluntary force of the knee extensors (Graven-Nielsen et al. 2002). Furthermore, partial blockade of group III and IV muscle afferents at the spinal level results in the attenuation of perceived fatigue, and increases central motor drive (Amann et al. 2009). Based on these findings, it is suggested that the increased activation of group III and IV afferents inhibits central motor drive and the ability to recruit motor units (Amann et al. 2011; Hureau et al. 2019).

A challenge in studying the fatigue–pain relationship (Mauger 2013; Pollak et al. 2014) is that most experimental pain-induction methods are notably different in their processing and response compared with the transmission and experience of EIP [i.e. differences in the neurological processes that result in the perception of pain, from transduction to perception (Olesen et al. 2012)]. For example, ischemic, electrical and thermal pain induction are experimental pain models that are non-specific to the muscle, and can also induce the perception of cutaneous pain (Staahl and Drewes 2004; Olesen et al. 2012). The additional stimulation of these superficial tissues can produce a subjective pain quality described as “sharp” or “stabbing” as opposed to the “aching” or “cramping” nature of muscle pain (Mense 1993). As such their use may be inappropriate in the investigation of EIP.

Consequently, an experimental model that induces muscle pain that feels like naturally occurring EIP and allows its contribution to fatigue to be investigated by decoupling EIP from exercise intensity is desirable. The intramuscular injection of hypertonic saline is a well-established and safe experimental method that, under resting conditions, induces standardised and reproducible acute pain often described as ‘aching’ and ‘cramping’ (Graven-Nielsen et al. 1997a, b, c). When injected, this solution activates predominantly group IV afferents with some contribution from myelinated group III nerve fibres (Laursen et al. 1999), which is similar to the nociceptive pathway of EIP (O’Connor and Cook 1999).

However, whilst hypertonic saline is established for inducing muscle pain, there has been limited comparison with the experience of EIP and minimal application to explore the fatigue–pain relationship. Indeed, in this field hypertonic saline is most widely used to investigate putative pain-induced changes to motor control (Hodges and Tucker 2011), maximal voluntary contraction (Graven-Nielsen et al. 2002), and high-intensity, short-duration exercise performance (Graven-Nielsen et al. 1997d) rather than its impact on exercise-induced fatigue. In addition, the exercise intensities, durations, and muscle groups used in these studies have limited relevance to exercise conditions where the impact of EIP on fatigue is most prominent [i.e. prolonged duration (> 2 min), exhaustive exercise in large, primary muscle groups involved in locomotive exercise] (Cook et al. 1997; Abbiss and Laursen 2008).

Therefore, the aims of this study were to (i) compare the qualitative experience (based on the total and subclass scores from the McGill Pain Questionnaire) of naturally occurring EIP to the pain elicited from an intramuscular injection of hypertonic saline into a locomotor muscle; and (ii) identify the effects of the muscle pain elicited by this method on the performance time of an endurance exercise task. We tested the hypothesis that the addition of an intramuscular injection of 5.8% hypertonic saline into the vastus lateralis (VL) to low-intensity exercise (i) produces a similar quality of pain (as defined by the McGill Pain Questionnaire) compared to naturally occurring EIP caused by a higher exercise intensity; and (ii) results in a shorter time to task failure compared to placebo and control conditions.

## Methods

### Ethical approval

The School of Sport and Exercises (University of Kent) Research Ethics Advisory Group (Prop 84_2016_17) approved all procedures and protocols in accordance with the Declaration of Helsinki. Written informed consent was gained from the participants prior to participation.

### Participants

Eighteen healthy and recreationally active participants (11 male, 7 female; mean ± SD: age, 24.5 ± 4.0 years; height 1.76 ± 0.1 m; body mass 73.9 ± 13.4 kg; physical activity 5.5 ± 2.3 h·w^−1^) volunteered to participate in the present study. The sample size was estimated based on the effect size reported in a similar exercise and pain study (Deschamps et al. 2014) to satisfy statistical power at 80%. All participants attended each visit in a similar psychological state as assessed by the Positive and Negative Affect Schedule (PANAS) (Watson et al. 1988), which was completed at the start of each visit.

Participants with existing knee pain, cardiorespiratory disease, neurological disorders, blood-borne viruses (e.g. hepatitis B/C and HIV), sore deep tissues, phobia to needles and any allergy were excluded from the study. Participants consuming supplements or medications that alter pain perception during the course of the study were also excluded. Before each visit, participants were instructed to refrain from vigorous exercise (24 h) and abstain from the consumption of alcohol (48 h), analgesics (6 h) and caffeine (8 h).

### Experimental procedures

Participants attended the laboratory on five occasions, with each visit separated by 2–7 days. In the initial visit, anthropometric measures were recorded, and participants were familiarised with all measures relating to the experimental protocol, including a practice of knee extensor maximal voluntary contractions (MVCs). Five minutes after the MVCs, participants performed an isometric time to task failure (TTF) at 20% maximal voluntary torque (MVT). In visit 2, participants received a single injection of hypertonic saline (Rest HYP), whilst seated at rest (see “Intramuscular injection procedure”). Upon the completion of the injection, participants were asked to continuously rate muscle pain intensity, with the visit concluding once the participant had returned to the state of ‘no pain’. In a further three visits (visits 3–5), participants performed a TTF at 10% MVT in three conditions in the presence of no injection (10% MVT, control), isotonic saline (10% MVT + ISO, placebo) and hypertonic saline (10% MVT + HYP). In the 10% MVT + ISO and 10% MVT + HYP visits, an intramuscular injection was administered prior to the TTF, with the task commencing within 3 s of needle removal. Conditions were performed in a single-blind, randomised and counter-balanced order.

### Time to task failure (TTF) protocol

All visits were performed seated on an isokinetic dynamometer (Cybex HUMAC Norm isokinetic dynamometer; CSMi, Soughton, MA, USA), set up for the right leg with knee angle at 75° flexion (0° = full extension of the knee), and a hip angle at 90°. At the start of each visit, participants completed a 5 min self-paced, submaximal warm-up on a cycle ergometer (Wattbike Ltd, Nottingham, UK) followed by 3 × 3 s MVCs separated by 90 s rest. The highest torque produced across the three MVCs was defined as the MVT. The TTF commenced 5 min after the MVCs, with the participants directed to maintain a submaximal isometric contraction of the right knee extensors. The participants received visual feedback of the target torque on a computer screen but were unaware of the overall time elapsed. The task was limited to a maximum of 20 min, or was terminated when the torque fell below the target for more than 3 s. Within 3 s of task cessation participants performed a final MVC.

### Intramuscular injection procedure

A single bolus of 1.0 mL 5.8% hypertonic saline was injected in the VL (middle third of the lateral aspect of the thigh) of the right leg to induce acute muscle pain. Injection of a single bolus of 1.0 mL 0.9% isotonic saline was implemented as a placebo. The injection was performed manually in a 20 s window (10 s infusion period) using a 3-mL Luer-Lok syringe connected to a 25 G × 38 mm SurGuard2 disposable stainless needle (Terumo, Japan).

### Perceptual measurements

At the start of each visit, participants were asked to rate (on a visual analogue scale) how much pain they expected to experience (anchored to the non-injury pain experienced during exercise) (0 = “no pain” to 10 = “worst possible pain”) and their confidence to cope with the expected level of pain (0 = “not confident at all” to 10 = “completely confident”). This provides a measure of pain-specific self-efficacy which is believed to a predictor of pain tolerance and endurance (Motl et al. 2007; Schmitz et al. 2013). Two characteristics of pain were evaluated: intensity and quality. During all visits, pain intensity was continuously scored on a moment-to-moment basis using an electronic visual analogue scale (VAS) ranging from 0 (“no pain”) to 10 (“extremely intense pain”) (Cook et al. 1997) and anchored to previous experiences of naturally occurring EIP (Astokorki and Mauger 2017). The device automatically sampled and recorded the reported pain intensity every 5 s, which allowed for values such as VAS onset (the time-point at which the stimulus is first perceived to be greater than “no pain”) peak pain intensity (VAS peak), time to maximal intensity (from the commencement of sampling), mean pain intensity (the mean VAS from the commencement of sampling until task failure), duration of pain (from VAS onset until the state of “no pain”), and VAS area (area under VAS curve) to be calculated.

The quality of pain was established by the long-form McGill Pain Questionnaire (MPQ) (Melzack 1975) which contains a total of 20 categories of adjectives describing four major subclasses of pain experience (sensory, affective, evaluative and miscellaneous). Each category contains between two and six similar adjectives arranged in ascending order of implied pain intensity and is assigned rank value based on this order (e.g. the descriptor associated with the least pain within the category is assigned a value of 1). Participants were permitted to select a maximum of one word per category (should any of the descriptors apply). The descriptors chosen by the participants were subsequently summed to calculate scores for each subclass (Subclass Rating Index) and the total score of all subclasses (Total Pain Rating Index), with the overall quality of pain expressed by descriptors chosen by more than one-third of participants. The MPQ was completed after the post-TTF MVC in each visit, and the return to “no pain” in the Rest HYP visit.

During all of the TTF trials at 10% MVT (visits 3–5), participants also reported Rating of Perceived Exertion (RPE), defined as the effort to drive the limb (Pageaux et al. 2015), using the 15-point Borg (6–20) scale (Borg 1998) every 30 s. Rating of Fatigue, the perceived inability of the muscle to produce torque, was recorded every 30 s for the first min, and every 60 s thereafter using the 11-point Rating of Fatigue (ROF) scale (Micklewright et al. 2017).

### Physiological measurements

During the TTFs at 10% MVT (visits 3–5) heart rate (HR) was recorded every 30 s using a Polar FT1 HR monitor paired with a coded T34 transmitter (Polar, Polar Electro, Kempele, Finland), and muscle electrical activity was continuously recorded using surface electromyography (sEMG). sEMG was acquired with square surface electrodes (Ag/AgCl, 32 × 32 mm; Nessler Medizintechnik, Innsbruck, Austria) mounted in a bipolar setup on skin which was shaven and cleansed with an alcohol swab. Electrodes were placed over the muscle belly of the VL, rectus femoris (RF) and vastus medialis (VM) in the direction of the muscle fibres, with a reference electrode placed on the patella. The electrical signal was sampled at 2000 Hz (Biopac MP150, Biopac Systems Inc., California, USA) and acquired in Spike2 software (Version 7; Cambridge Electronic Design).

The sEMG data were analysed using custom code written in MATLAB R2018a (The MathWorks, Massachusetts, USA). To create a linear envelope representation of the data, the raw sEMG signals were rectified by taking the absolute values, and two-pass zero-lag filtered using a fourth-order low-pass Butterworth filter with a cut-off frequency of 5 Hz. To analyse changes over time, the signals were divided into 10 s epochs. The mean sEMG amplitude for the VL, RF and VM over each 10 s epoch was extracted and normalised to the maximum sEMG amplitude of the prior MVCs (% MVC).

### Statistical analysis

All data are presented in the form of mean ± standard deviation (SD). Prior to statistical analysis, all data were checked for the assumptions associated with a paired samples *t* test, a one-way ANOVA and a repeated measures ANOVA as appropriate. Data that did not satisfy the Shaprio–Wilk test of normality (*P* < 0.05) were logarithmically transformed. The Bonferroni post-hoc correction was applied where appropriate. Cohen’s *d* and partial eta square (*ƞ*_*p*_^2^) values are reported as measures of effect size (Cohen 1988).

Due to between subject variability in TTF, an ‘individual iso-time’ approach as outlined by Nicolò et al. (2019) was applied to compare perceptual (pain intensity, RPE, ROF) and physiological (HR, sEMG) variables. The shortest TTF for each participant was used to identify four (RPE, ROF, HR) and ten (pain intensity and sEMG) time-points in which the three conditions were segmented. This approach maintains a majority of the time-series data (i.e. allows for the inclusion of all repeated recordings such as pain, RPE and ROF to be included) and provides a consistent number of data points to allow comparison between participants for all stated variables across the varying TTF times.

A two-way ANOVA with treatment factor with three fixed levels (10% MVT, 10% MVT + ISO, 10% MVT + HYP) and a repeated measures time factor with 10 time-points were used to test the effect of condition and time on pain intensity and sEMG during the TTF. Two-way ANOVAs with a treatment factor with three fixed levels (10% MVT, 10% MVT + ISO, Experimental) and a repeated measures time factor with 4 time-points were used for measures of RPE, ROF and HR recorded during the TTF. When an interaction effect was observed, post-hoc paired sample *t* tests were implemented to evaluate differences between conditions. Statistical significance was accepted at an alpha level of *P* < 0.05 except where a Bonferroni correction was applied (adjusted, *P* < 0.0042). All statistics were performed using SPSS Statistics v24.0 (SPSS, IBM, New York, USA).

## Results

As the TTF task was limited to a maximum of 20 min, participants that met this cut-off in any condition did not reach task failure or ‘exhaustion’, which does not provide a true indication of endurance performance. To account for this, these participants (*n* = 6) were subsequently removed from the dataset, and analysis was performed on the subset of participants (*n* = 12).

### Comparison of pain intensity and quality

Mean TTF at 20% MVT was 193 ± 50 s. As shown in Table 1, paired samples *t* tests revealed a significant difference in VAS scores between pain intensity during 20% MVT TTF and experimental muscle pain from rest HYP (*P* < 0.05). The 20% MVT task induced a significantly greater mean VAS, equivalent to between “somewhat strong” and “strong” pain intensity (*t*_11_ = 5.3, *P* < 0.001, CI_.95_ 1.1, 2.6, *d* = 1.8), which peaked after a longer period of time (*t*_11_ = 5.6, *P* < 0.001, CI_.95_ 64, 147, *d* = 1.7) and lasted for a shorter duration (*t*_11_ = − 3.9, *P* = 0.002, CI_.95_ − 175, − 49, *d* = 1.7) than the experimental muscle pain experienced in rest HYP.

**Table 1:** Summary VAS scores from 20% MVT, Rest HYP, 10% MVT, 10% MVT + ISO, 10% MVT + HYP TTF

	20% MVT	Rest HYP	10% MVT	10% MVT + ISO	10% MVT + HYP
VAS onset (s)	25 ± 22	7 ± 16	55 ± 36^**^	42 ± 29^*^	10 ± 9^*^
VAS mean	4.8 ± 1.0	3.0 ± 1.0^**^	5.3 ± 1.4	5.5 ± 1.2	6.3 ± 1.7^*†^
VAS peak	9.7 ± 0.7	5.8 ± 2.1^**^	9.5 ± 0.8	9.0 ± 1.5	9.2 ± 1.6
VAS time to peak (s)	181 ± 51	75 ± 31^**^	438 ± 171^**^	516 ± 282^**^	379 ± 229^*^
VAS duration (s)	168 ± 42	281 ± 84^*^	459 ± 185^**^	555 ± 270^**^	438 ± 241^*^
VAS area	899 ± 315	869 ± 386	2713 ± 1282^**^	3248 ± 1493^**^	2740 ± 1521^*^

Values are means ± SD

^*^Significantly different *vs* 20% MVT (*P* < 0.05)

^**^Significantly different *vs* 20% MVT (*P* < 0.001)

^†^Significantly different *vs* 10% MVT (*P* < 0.05)

Differences in VAS scores were also reported between 20% TTF and the TTFs at 10% MVT (*P* < 0.05). The VAS onset was significantly slower in 10% MVT (*t*_11_ = − 5.0, *P* < 0.001, CI_.95_ − 44, − 17, *d* = 1.0) and 10% MVT + ISO (*t*_11_ = − 2.3, *P* = 0.043, CI_.95_ − 33, − 1, *d* = 0.7), with a quicker onset in 10% MVT + HYP (*t*_11_ = 2.2, *P* = 0.0047, CI_.95_ 0.2, 29, *d* = 0.9). A greater VAS mean, equivalent to between “strong” and “very strong” pain, was observed in the 10% MVT + HYP condition compared to 20% MVT (*t*_11_ = − 2.8, *P* = 0.017, CI_.95_ − 2.6, − 0.3, *d* = 1.1) and 10% MVT (*t*_11_ = − 2.3, *P* = 0.044, CI_.95_ − 1.97, − 0.03, *d* = 0.6).

The VAS in all three conditions performed at 10% MVT peaked after a longer period of time (10% MVT; *t*_11_ = − 6.5, *P* < 0.001, CI_.95_ − 344, − 170, *d* = 2.0, 10% MVT + ISO; *t*_11_ = − 4.9, *P* < 0.001, CI_.95_ − 484, − 185, *d* = 1.7, 10% MVT + HYP; *t*_11_ = − 3.5, *P* = 0.005, CI_.95_ − 321, − 74, *d* = 1.2) and lasted longer in duration (10% MVT; *t*_11_ = − 6.3, *P* < 0.001, CI_.95_ − 394, − 189, *d* = 2.2, 10% MVT + ISO; *t*_11_ = − 5.6, *P* < 0.001, CI_.95_ − 538, − 234, *d* = 2.0, 10% MVT + HYP; *t*_11_ = − 4.2, *P* = 0.001, CI_.95_ − 411, − 130, *d* = 1.6) than the 20% MVT condition. This contributed to a greater VAS area (10% MVT; *t*_11_ = − 5.4, *P* < 0.001, CI_.95_ − 2551, − 1077, *d* = 1.9, 10% MVT + ISO; *t*_11_ = − 5.9, *P* < 0.001, CI_.95_ − 3233, − 1466, *d* = 2.2, 10% MVT + HYP; *t*_11_ = − 4.4, *P* = 0.001, CI_.95_ − 2754, − 929, *d* = 1.7) in the 10% MVT conditions compared to 20% MVT.

Overall, as shown in Table 2, the dimensional quality of pain experienced during 20% MVT was similar to rest HYP for the sensory (*P* = 0.123) and miscellaneous (*P* = 0.189) dimensions, but not for the affective (*P* = 0.008) and evaluative (*P* = 0.007) subclasses. The 20% MVT task produced a greater mean Total Pain Index of 30 ± 11 (*t*_11_ = 2.9, *P* = 0.016, CI_.95_ 2, 18, *d* = 0.7) than rest HYP (20 ± 9), and as shown in Table 2, and was defined by descriptives representing all dimensions in the MPQ. However, the 10% MVT + HYP condition, with a mean total pain index of 29 ± 14, produced a similar overall subjective quality of pain to 20% MVT (*t*_11_ = 0.3, *P* = 0.743, CI_.95_ − 6, 8, *d* = 0.1). Paired samples * t* tests revealed no significant difference in Subclass Rating Index between 10% MVT + HYP and 10% MVT (Sensory; *P* = 0.704, Affective: *P* = 0.429, Evaluative; *P* = 0.878; Miscellaneous, *P* = 0.410) as well as 10% MVT + HYP and 20% MVT (Sensory; *P* = 0.941, Affective: *P* = 0.394, Evaluative; *P* = 0.504; Miscellaneous, *P* = 0.810) for all classifications (Table 2).

**Table 2:** Frequently selected words from the MPQ subclasses

Subclass		20% MVT	REST HYP	10% MVT	10% MVT + ISO	10% MVT + HYP
		Throbbing (33%)	Throbbing (50%)	Lacerating (33%)	Throbbing (50%)	Throbbing (58%)
		Sharp (58%)	Shooting (42%)	Cramping (58%)	Cramping (41%)	Drilling (33%)
		Cramping (33%)	Sharp (33%)	Pulling (33%)	Burning (50%)	Cramping (67%)
		Pulling (33%)	Cramping (67%)	Searing (33%)	Aching (67%)	Burning (42%)
		Hot (33%)	Aching (67%)	Aching (50%)		Aching (50%)
		Burning (33%)	Tender (33%)			Heavy (33%)
		Hurting (33%)				
Sensory		Aching (58%)				
	SRI	18 ± 6	15 ± 6	18 ± 9	15 ± 6	18 ± 9
Affective		Exhausting (50%)		Exhausting (75%)	Tiring (33%) Gruelling (33%)	Tiring (42%) Exhausting (42%) Gruelling (33%)
	SRI	3 ± 3	1 ± 1^*^	3 ± 2	2 ± 2	3 ± 2
Evaluative		Intense (50%)	Intense (33%)	Intense (58%)	Intense (67%)	Intense (67%)
	SRI	4 ± 2	2 ± 2^*^	3 ± 2	3 ± 1	3 ± 1
Miscellaneous		Radiating (33%) Tight (33%)	Radiating (33%)			
	SRI	5 ± 4	3 ± 3	4 ± 3	5 ± 4	5 ± 4
	PRI(T)	30 ± 11	20 ± 9^*^	28 ± 12	26 ± 11	29 ± 14

The frequently selected words from the MPQ are shown with the percentage of participants that selected these words. Data on Subclass Rating Index (SRI) and Pain Rating Index (Total) presented as Mean ± SD

^*^Significantly different *vs* 20% MVT (*P* < 0.05)

### Time to task failure (TTF)

**Fig. 1:**
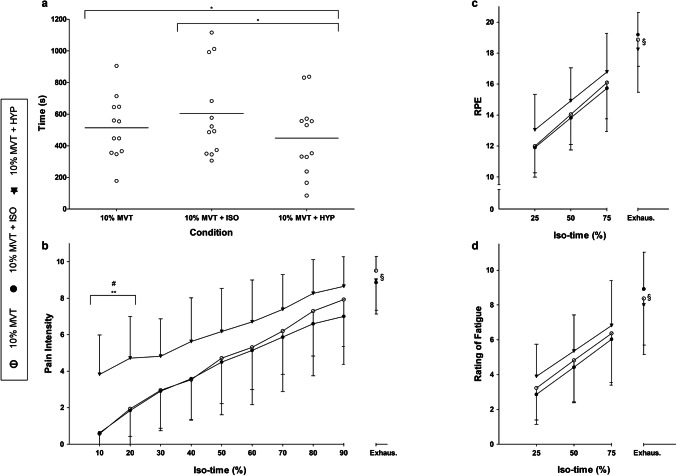
Performance and perceptual differences between conditions. Differences in TTF between conditions (**a**), and pain intensity (**b**) and RPE (**c**) and ROF (**d**) over iso-time between conditions during the TTF. *Significant difference between conditions (*P* < 0.05). **Significant difference between 10% MVT + HYP and 10% MVT (*P* ≤ 0.001). #Significant difference between 10% MVT + HYP and 10% MVT + ISO (*P* < 0.001). §Significant main effect of iso-time

An ANOVA revealed a significant difference between conditions (*F*_2,22_ = 6.7, *P* = 0.005, *ƞ*_*p*_^2^ = 0.378) with 10% MVT + HYP causing a significantly (*t*_11_ = 3.4, *P* = 0.006, CI_.95_ 55, 257, *d* = 0.6) shorter TTF (448 ± 240 s) compared to both 10% MVT + ISO (605 ± 285 s), and 10% MVT (514 ± 197 s) (*t*_11_ = 2.3, *P* = 0.040, CI_.95_ 4, 127, *d* = 0.3) (Fig. 1a). No significant differences were observed between 10% MVT and 10% MVT + ISO (*t*_11_ = − 1.8, *P* = 0.104, CI_.95_ − 204, 22 *d* = 0.4).

Paired samples *t* tests showed that post-TTF MVT significantly decreased in 10% MVT (pre = 304 ± 56 N.m, post = 191 ± 62 N.m), 10% MVT + ISO (pre = 300 ± 62 N.m, post = 197 ± 64 N.m) and 10% MVT + HYP (pre = 308 ± 65 N.m, post = 187 ± 66 N.m) in comparison to pre-TTF MVT (*P* < 0.001). No significant difference was observed between conditions for absolute decrement in MVT (*F*_2,22_ = 1.0, *P* = 0.379, *ƞ*_*p*_^2^ = 0.204). An ANOVA also demonstrated no significant difference between conditions for positive affect (*F*_2,22_ = 1.8, *P* = 0.189, *ƞ*_*p*_^2^ = 0.141), and negative affect (*F*_2,22_ = 1.4, *P* = 0.263, *ƞ*_*p*_^2^ = 0.114) recorded prior to the TTF.

### Pain intensity

An ANOVA revealed a significant difference in pain expectations between conditions (*F*_2,22_ = 9.6, *P* = 0.001, *ƞ*_*p*_^2^ = 0.467), but not in confidence to cope with the expected pain (*F*_2,22_ = 2.3, *P* = 0.125, *ƞ*_*p*_^2^ = 0.172). Subsequent pairwise comparisons found greater expectations of pain in 10% MVT + ISO (7.2 ± 1.9) (*t*_11_ = − 3.8, *P* = 0.003, CI_.95_ − 2, − 1, *d* = 0.7) and 10% MVT + HYP (7.5 ± 1.3)(*t*_11_ = − 4.5, *P* = 0.001, CI_.95_ − 2, − 1, *d* = 1.0) compared to 10% MVT (6.0 ± 1.6) with no significant difference between 10% MVT + ISO and 10% MVT + HYP (*t*_11_ = − 0.7, *P* = 0.518, CI_.95_ − 1, 1, *d* = 0.2).

The 3 × 10 (condition × iso-time) repeated measures ANOVA highlighted a significant effect of condition (*F*_2,22_ = 6.5, *P* = 0.006, *ƞ*_*p*_^2^ = 0.372) and iso-time (*F*_2.8,31.2_ = 82.2, *P* < 0.001, *ƞ*_*p*_^2^ = 0.882) for perceived pain during the TTF (Fig. 1b). A significant interaction effect for pain over iso-time between conditions during the TTF was observed (*F*_3.9,42.4_ = 3.4, *P* = 0.018, *ƞ*_*p*_^2^ = 0.236). Follow-up targeted paired-sample *t* tests with a Bonferroni correction revealed a significantly greater VAS pain intensity at 10% iso-time (43 ± 21 s) in 10% MVT + HYP compared to both 10% MVT (*t*_11_ = − 6.4, *P* < 0.001, CI_.95_ − 43.7, − 21.3, *d* = 1.9) and 10% MVT + ISO (*t*_11_ = − 5.8, *P* < 0.001, CI_.95_ − 44.2, − 19.9, *d* = 1.9) and at 20% iso-time (86 ± 42 s) in contrast with 10% MVT (*t*_11_ = − 4.3, *P* = 0.001, CI_.95_ − 42.1, − 13.4, *d* = 1.3) and 10% MVT + ISO (*t*_11_ = − 6.3, *P* < 0.001, CI_.95_ − 38.9, − 18.6, *d* = 1.5).

### Perceptual measurements

The 3 × 4 (condition × iso-time) repeated measures ANOVA revealed no significant main effect of condition for ROF or RPE (*P* > 0.05). Both ROF and RPE had a significant effect of iso-time (ROF; *F*_1.4,15.9_ = 104.1, *P* < 0.001, *ƞ*_*p*_^2^ = 0.904, RPE; *F*_1.5,16.3_ = 87.8, *P* < 0.001, *ƞ*_*p*_^2^ = 0.889), and an interaction effect (ROF; *F*_2.1,23.4_ = 6.9, *P* = 0.004, *ƞ*_*p*_^2^ = 0.387, RPE; *F*_2.8,31.1_ = 4.6, *P* = 0.010, *ƞ*_*p*_^2^ = 0.296) (Fig. 1c, d). Followup paired samples *t* tests with a Bonferroni correction (*P* > 0.004) revealed no significant differences at any iso-time point between conditions for both ROF and RPE.

### Surface electromyography (sEMG)

**Fig. 2:**
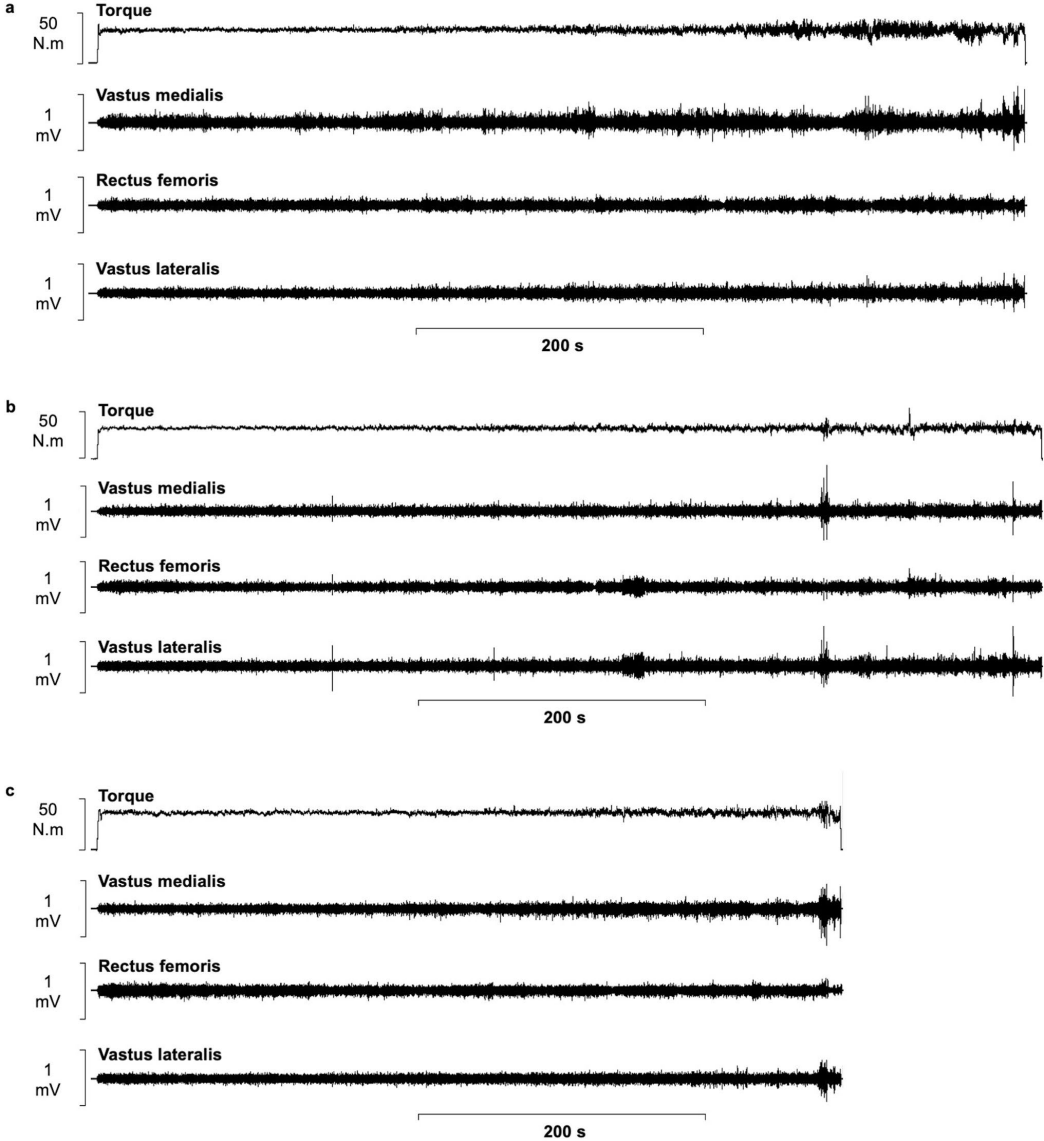
Torque and sEMG data during the TTF of the 10% MVT (**a**), 10% MVT + ISO (**b**) and 10% MVT + HYP (**c**) conditions for a representative participant. The TTF was significantly shortened in the 10% MVT + HYP condition

**Fig. 3:**
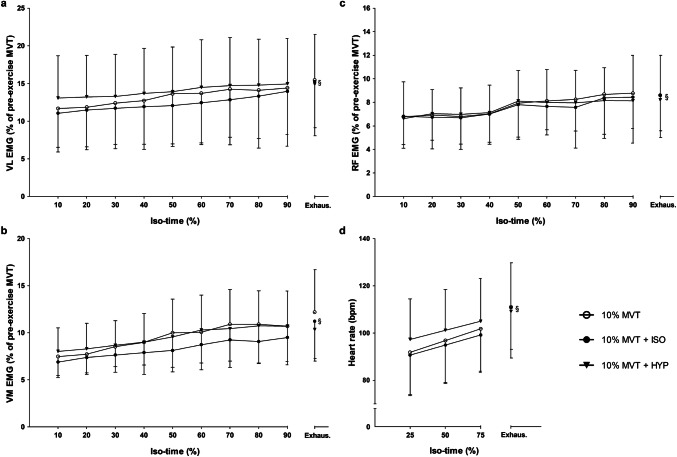
Physiological differences between conditions. EMG of the VL (**a**), VM (**b**) and RF (**c**) over iso-time between conditions during the TTF. HR differences between conditions over iso-time during the TTF (**d**). §Significant main effect of iso-time (*P* < 0.05)

Due to a loss in sEMG signal, two participants were removed from the dataset and analysis was performed on the remaining participants (*n* = 10). A 3 × 10 (condition × iso-time) repeated measures ANOVA demonstrated no significant main effect of condition in either the VL (*F*_2,18_ = 1.3, *P* = 0.288, *ƞ*_*p*_^2^ = 0.129), VM (*F*_2,18_ = 1.9, *P* = 0.174, *ƞ*_*p*_^*2*^ = 0.177) or RF (*F*_2,18_ = 0.5, *P* = 0.613, *ƞ*_*p*_^2^ = 0.053). A significant effect of iso-time in the activity of the VL (*F*_1.5,13.2_ = 19.3, *P* < 0.001, *ƞ*_*p*_^2^ = 0.682), VM (*F*_1.8,16.4_ = 14.2, *P* < 0.001, *ƞ*_*p*_^2^ = 0.612), and RF (*F*_2.0,18.2_ = 6.7, *P* = 0.007, *ƞ*_*p*_^2^ = 0.426) was reported (Fig. 2). There was no interaction effect observed in the RF (*F*_18,162_ = 0.4, *P* = 0.994, *ƞ*_*p*_^2^ = 0.037). A significant interaction effect was reported for VL (*F*_18,162_ = 3.5, *P* < 0.001, *ƞ*_*p*_^2^ = 0.278) and VM (*F*_18,162_ = 2.2, *P* = 0.006, *ƞ*_*p*_^2^ = 0.195) activity over iso-time between conditions; however, subsequent followup-targeted paired sample *t* tests with a Bonferroni correction demonstrated no significant differences (Fig. 3a–c).

### Heart rate (HR)

The 3 × 4 (condition × iso-time) repeated measures ANOVA revealed no significant main effect of condition (*F*_1.3,14.1_ = 0.8, *P* = 0.404, *ƞ*_*p*_^2^ = 0.071). There was a significant effect of iso-time (*F*_1.1,12.3_ = 39.6, *P* < 0.001, *ƞ*_*p*_^2^ = 0.783), and an interaction effect for HR and iso-time between conditions during the TTF (*F*_1.7,18.9_ = 6.0, *P* = 0.012, *ƞ*_*p*_^2^ = 0.352). Subsequent paired samples *t* tests with a Bonferroni correction revealed no significant differences between conditions (Fig. 3d).

## Discussion

This study confirms that the pain experienced during knee extensor exercise at 10% MVT can be made to feel like that of a higher exercise intensity, through the intramuscular injection of hypertonic saline into the VL. Using this intervention, exercise-induced fatigue occurred more rapidly, with participants reaching task failure earlier when exercising with a greater pain intensity (Fig. 1b). This study therefore provides indicative evidence to support the notion that pain is a significant factor affecting endurance exercise performance.

### Hypertonic saline combined with light exercise feels like EIP

The novel question the present study strived to answer was whether the addition of hypertonic saline to light intensity exercise at 10% MVT produces an elevated pain intensity which also feels similar to the naturally occurring EIP during a higher exercise intensity (20% MVT). Thus, the first key finding from this study is that when combined with light exercise (10% MVT), the hypertonic saline induced a descriptive quality of pain similar to the EIP from both the 10% and 20% MVT exercise tasks (but with a higher intensity). This is in contrast to the administration of hypertonic saline at rest, where our findings were consistent with the established literature — a ‘moderate’ to ‘somewhat strong’ pain, described as ‘cramping’, ‘aching’, ‘throbbing’ and ‘intense’ (Graven-Nielsen et al. 1997a, b, c). Furthermore, in these resting conditions, whilst the sensory and miscellaneous quality of experimental pain was similar to the naturally occurring EIP experienced during the 20% MVT task, there were differences in pain intensity and quality. In particular, the 20% MVT task produced a higher pain intensity that was also described in the affective (e.g. ‘exhausting’) dimension. This suggests that for hypertonic saline to induce a pain that feels like EIP, it needs to be combined with at least light intensity exercise. When this was done, participants experienced an elevated overall intensity of pain (compared to both 10% and 20% MVT) but were unable to distinguish between the experimental muscle pain produced by the hypertonic saline and the EIP from the muscular contraction. The findings of this study therefore provide support for this hypertonic saline model for uncoupling the exercise intensity and EIP relationship (Cook et al. 1997) — i.e. causing a light exercise intensity to feel like a harder exercise intensity.

### Effect of pain on isometric TTF

The present study demonstrates that greater levels of pain in a fresh, undamaged, large locomotor muscle group significantly shortens TTF during an isometric endurance task. Indeed, TTF was significantly shorter in the 10% MVT + HYP condition than both the 10% MVT and 10% MVT + ISO conditions, with an impaired performance of 12 to 26%. As all conditions were performed at the same intensity (10% MVT) and with participants in a similar psychological state, these differences in TTF can be attributed solely to increasing the experience of pain in the 10% MVT + HYP condition, as clearly shown in Fig. 1b.

Previous research that has used hypertonic saline to induce muscle pain have predominantly applied it in smaller muscles or muscle groups (e.g. biceps brachii, tibialis anterior and gastrocnemius (Graven-Nielsen et al. 1997d; Ciubotariu et al. 2004; Khan et al. 2011)) and have not focused on producing a pain experience that feels like EIP. The VL is a large muscle with a key role in the generation of force during basic locomotor tasks (e.g. walking, stair climbing) and contributes to propulsive energy during cycling (Raasch et al. 1997), as well as the stance and swing phase in running (Sasaki and Neptune 2006). Understanding the effects of an increased overall pain experience in this muscle (and surrounding knee extensor group) at a contraction intensity utilised during cycling exercise (Löllgen et al. 1980) therefore provides information that closely translates to exercise performance and a clinical context. Care should however be taken when extrapolating findings to whole-body exercise or dynamic contraction.

During the impaired TTF performance in the 10% MVT + HYP condition, pain intensity was significantly elevated in the first 20% of the task, with a continued linear increase until task failure. Indeed, the intensity of pain reported in the 10% MVT + HYP condition was elevated by approximately 3.3 at 10% iso-time and 2.8 at 20% iso-time on the VAS scale. The hypertonic saline in the 10% MVT + HYP condition would have increased the activation of the group III and IV nociceptive afferents in addition to the rapidly increasing noxious environment arising from the metabolites produced as a result of the exercise task (O’Connor and Cook 1999), which might explain the shorter TTF in the 10% MVT + HYP condition.

This explanation is in accordance with the “Sensory Tolerance Limit”, where in open-loop exercise tasks (i.e. TTF) the increased inhibitory feedback from Group III and IV afferents contributes to an individual and task-specific threshold, which when reached the exercise is voluntarily terminated (Amann and Dempsey 2008; Amann 2011). With similar values for RPE and ROF between conditions, it is likely the elevated pain intensity during the TTF at 10% MVT + HYP resulted in this sensory tolerance limit being reached sooner, causing a faster occurrence of task failure compared to the 10% MVT and 10% MVT + ISO conditions (Aboodarda et al. 2020).

In addition, the increased nociceptive activity (a specific type of afferent feedback) may have limited central motor drive and voluntary activation of the knee extensors (Amann et al. 2009, 2011; Aboodarda et al. 2020), a notion which is supported by evidence showing a relationship between group III and IV muscle afferents and neuromuscular fatigue (Amann et al. 2015; Sidhu et al. 2018). In support of this, Henriksen and colleagues (2011) reported a reduced capacity of the knee extensors to produce an MVT in the presence of pain. Furthermore, findings from Graven-Nielsen and colleagues (2002) demonstrated that experimental muscle pain (from the hypertonic saline model) reduces MVT despite an unaffected twitch torque, implying that performance decrements were due to mechanisms residing in the central nervous system rather than the peripheral musculature (Graven-Nielsen et al. 2002).

Rather than a uniform inhibitory/facilitatory effect on agonist and antagonist muscle activity (Pain Adaptation Model, Lund et al. 1991), it is now recognised that pain does not cause uniform inhibition/excitation effects across the motor neurone pool, but instead causes a redistribution of activity within and between muscles (Hodges and Tucker 2011). Accordingly, the decreased performance caused by the overall increased pain experience in the current study could also be explained by a slight change in the direction of knee extensor torque to a more lateral/medial plane (Tucker and Hodges 2010). In this context, the gross feature of the task would remain (i.e. knee extension), but the efficiency of this movement would be compromised. Motor unit recruitment order, or a recruitment of larger units at lower torques, could have also affected the task performance. In an endurance task lasting several minutes, the preferential recruitment of large high threshold motor units (which may include Type II muscle fibres) above low threshold small motor units (Type I muscle fibres) would likely have consequences for the rate at which fatigue occurs (both metabolic and neural), leading to a shorter TTF (Edwards 1981). Whilst not observed in the present study, an increase in sEMG would be indicative of an increased central drive to the muscle and/or an increased recruitment of high threshold motor units (Gerdle et al. 2000), which would be in line with Hodges and Tucker’s “moving differently in pain” theory (Hodges and Tucker 2011).

### Methodological considerations

The methods used in this study preclude the ability to identify which, or combination of these mechanisms, may have contributed to the shorter TTF. Indeed, combinations of peripheral nerve/transcranial stimulation, multiple force transducers, and fine wire electrodes would be required for this. In addition, the sensitivity of the sEMG setup in the present study did not allow for the detection of non-uniform changes across the motor neurone pool (i.e. any alterations are unlikely to be discovered with bipolar sEMG). As such, an approach that allows for the identification of individual motor units would be more appropriate for the observation of subtle changes in activity within and between the muscles (i.e. high-density EMG). Differential responses to pain between male and female participants are also acknowledged, with the present study not accounting for or attempting to control the menstrual cycle of the female participants. Indeed, hormonal changes across the different phases of the menstrual cycle may cause some difference in pain perception to experimental pain (Sherman and LeResche 2006).

## Conclusion

The injection of hypertonic saline into the VL during a sustained low-intensity isometric contraction provides an overall qualitative experience of pain that feels like naturally occurring EIP induced by a higher intensity exercise. When applied to submaximal exercise, this additional pain caused a shorter TTF compared with a placebo and control condition. It is plausible that the mechanisms responsible for the shorter TTF were related to increased activity of group III and IV nociceptive afferents from the injected muscle. The present study therefore provides important evidence that muscle pain has a direct impact on endurance performance.

## Data Availability

The datasets generated during and/or analysed during the current study are available from the corresponding author on reasonable request.

## Abbreviations

ANOVA: Analysis of variance
EIP: Exercise-induced pain
HR: Heart rate
HYP: Hypertonic saline
ISO: Isotonic saline
MPQ: McGill Pain Questionnaire
MVC: Maximal voluntary contraction
MVT: Maximal voluntary torque
PANAS: Positive and negative affect schedule
PRI(T): Pain rating index (total)
RF: Rectus femoris
RPE: Rating of perceived exertion
SD: Standard deviation
sEMG: Surface electromyography
SRI: Subclass rating index
VAS: Visual analogue scale
VL: Vastus lateralis
VM: Vastus medialis
